# Antibiotic-Potentiating Activity of the *Schinus terebinthifolius* Raddi Essential Oil against MDR Bacterial Strains

**DOI:** 10.3390/plants12081587

**Published:** 2023-04-08

**Authors:** Maria Milene Costa da Silva, José Bezerra de Araújo Neto, Antonia Thassya Lucas dos Santos, Cícera Datiane de Morais Oliveira-Tintino, Ana Carolina Justino de Araújo, Priscilla Ramos Freitas, Luiz Everson da Silva, Wanderlei do Amaral, Cícero Deschamps, Francisco Roberto de Azevedo, Clara Mariana Gonçalves Lima, Nadezhda Golubkina, João Tavares Calixto-Júnior, Jaime Ribeiro-Filho, Henrique Douglas Melo Coutinho, Gianluca Caruso, Saulo Relison Tintino

**Affiliations:** 1 Department of Biological Sciences, Regional University of Cariri—URCA, Rua Cel. Antonio Luis 1161, Pimenta, Crato 63105-000, CE, Brazil; 2 Department of Biological Chemistry, Regional University of Cariri—URCA, Rua Cel. Antonio Luis 1161, Pimenta, Crato 63105-000, CE, Brazil; 3 Postgraduate Program in Sustainable Territorial Development, Coastal Sector, Federal University of Paraná, Curitiba 80060-000, PR, Brazil; 4 Centro de Ciencias Agrarias e da Biodiversidade, Federal University of Cariri—UFCA, Crato 63048-080, CE, Brazil; 5 Department of Food Science, Federal University of Lavras, Lavras 37203-202, MG, Brazil; 6 Federal Scientific Center of Vegetable Production, Selectsionnaya 14, VNIISSOK, Odintsovo District, 143072 Moscow, Russia; 7 Oswaldo Cruz Foundation (Fiocruz), Fiocruz Ceará, Eusébio 61773-270, CE, Brazil; 8 Department of Agricultural Sciences, University of Naples Federico II, 80055 Naples, Italy

**Keywords:** *Schinus terebinthifolius*, essential oil, antibacterial, antibiotic resistance

## Abstract

*Escherichia coli*, *Pseudomonas aeruginosa*, and *Staphylococcus aureus* are the primary bacteria that cause clinical infections, such as urinary and intestinal infections, pneumonia, endocarditis, and sepsis. Bacterial resistance is an innate natural occurrence in microorganisms, resulting from mutations or the lateral exchange of genetic material. This serves as evidence for the association between drug consumption and pathogen resistance. Evidence has demonstrated that the association between conventional antibiotics and natural products is a promising pharmacological strategy to overcome resistance mechanisms. Considering the large body of research demonstrating the significant antimicrobial activities of *Schinus terebinthifolius* Raddi, the present study aimed to evaluate the chemical composition and antibiotic-enhancing effects of *Schinus terebinthifolius* Raddi essential oil (STEO) against the standard and multidrug-resistant strains of *Escherichia coli*, *Pseudomonas aeruginosa*, and *Staphylococcus aureus*. The STEO was extracted by hydrodistillation using a Clevenger-type vacuum rotary evaporator. The Minimum Inhibitory Concentration (MIC) of the STEO was assessed by the microdilution method to evaluate the antibacterial activity. The antibiotic-enhancing activity of the essential oil was assessed by determining the MIC of antibiotics in the presence of a sub-inhibitory concentration (MIC/8) of the natural product. The GC-MS analysis revealed alpha-pinene (24.3%), gamma-muurolene (16.6%), and myrcene (13.7%) as major constituents of the STEO. The STEO potentiated the enhanced antibacterial activity of norfloxacin and gentamicin against all the strains and increased the action of penicillin against the Gram-negative strains. Therefore, it is concluded that although the STEO does not exhibit clinically effective antibacterial activity, its association with conventional antibiotics results in enhanced antibiotic activity.

## 1. Introduction

The discovery of antibiotics and their use against bacterial infections represented one of the most relevant therapeutic advances in the 20th century, significantly contributing to the increase in life expectancy and the rise of modern medicine [1,2,3]. However, the indiscriminate use and inappropriate prescription of antibiotics have contributed to the development of bacterial resistance [4,5], limiting the effectiveness of the currently available drugs, including last-generation antibiotics. The rapid escalation of bacteria that are immune to antibiotics has resulted in widespread concern within the global health community. These recurrent infections pose a significant challenge due to their difficulty to manage since they require expensive and toxic antibiotics that are hard to obtain. This situation has an impact on public health, ecology, and the economy. Recent research has indicated that over 2 million individuals in the United States are infected with antibiotic-resistant bacteria, leading to 23,000 deaths annually [6].

Bacterial resistance is a phenomenon that has been occurring frequently, resulting from mutations or the lateral exchange of genetic material. This serves as evidence for the association between drug consumption and pathogen resistance. A group of genes called “bacterial resistome” orchestrates the resistance profile. It is categorized as intrinsic resistance, which pertains to a collection of organisms that become resistant to certain antibiotics without receiving genetic material and could be obtained via mutations [7].

*Escherichia coli*, *Pseudomonas aeruginosa*, and *Staphylococcus aureus* are the primary bacteria that cause clinical infections, such as urinary and intestinal infections, pneumonia, endocarditis, and sepsis. *Escherichia coli* is one of the main bacteria that cause infectious diseases in humans. It is known by the production of enterotoxins, whose properties have been widely investigated. Its transmission can occur through ingesting contaminated food and water and has the human being as a secondary dissemination vehicle. It is also worth mentioning that pathogenic *E. coli* has some serotypes, which are determined based on pathogenicity, virulence, and the damage they can cause. Its transmission can occur through the ingestion of contaminated food and water and has the human being as a secondary dissemination vehicle. Eminent for being considered the cause of high morbidity and mortality rates, *E. coli* is not just a harmless commensal present in the intestinal tract since some types of harmful *E. coli* can cause a series of complications in the organism. Among these complications, diarrhea, dysentery, gastroenteritis, urinary tract infection, and meningitis are highlighted [8].

*Pseudomonas aeruginosa* is responsible for various infections affecting the skin, eyes, and ears. The broad distribution environment of *Pseudomonas* strains and the significant variety of enzymes and toxins that potentiate their virulence ensure their growth and transmission, commonly associated with burns and hospital infections in neonatal intensive care units. In immunocompromised patients, such as people with cystic fibrosis and advanced-stage HIV infections, there is a considerable risk of community-acquired contamination. This bacterium can fall into the category of multidrug-resistant (MDR), which means it is not susceptible to at least one agent in three or more antimicrobial categories. It can also be categorized as extensively resistant (XDR), meaning it is not susceptible to at least one agent in all but two or fewer antimicrobial categories, leaving only one or two categories in which the bacterial isolates remain susceptible. If the bacterial isolate is non-susceptible to all agents in all antimicrobial categories, it is categorized as pan-resistant (PDR), indicating that no agents tested are susceptible to that organism. Therefore, a bacterial isolate that is characterized as XDR is also characterized as MDR. Similarly, a bacterial isolate must be XDR to be classified as PDR [9].

*Staphylococcus aureus* is a significant causative agent of human and nosocomial infections. In addition to causing different types of poisoning, *S. aureus* is the most common etiological agent of purulent infections (e.g., boil, carbuncle, abscess, myocarditis, endocarditis, pneumonia, meningitis, arthritis bacterial). The transmission of this pathogen can result from the ingestion of contaminated water and food, either due to carelessness in handling it or inadequate refrigeration and cooking. When growing in environments with favorable development conditions, this microorganism produces toxins, such as enterotoxin, a substance that causes food poisoning. Moreover, *S. aureus* is an important cause of intestinal infections, folliculitis, puerperal mastitis, and boils, in addition to severe infections such as sepsis, septic shock, endocarditis, and pneumonia [7].

One of the main public health problems with *S. aureus*, in addition to its virulence, is its resistance. We emphasize the MRSA strains (methicillin-resistant *S. aureus*), are strains of this bacterium which have acquired resistance to methicillin and other beta-lactam antibiotics. According to the World Health Organization (WHO), MRSA is a major cause of healthcare-associated infections, and its prevalence is increasing worldwide [10]. Studies have shown that the emergence and spread of MRSA are associated with various risk factors, including prolonged hospitalization, invasive medical procedures, and exposure to antibiotics. Studies have shown that the emergence and spread of MRSA are associated with various risk factors, including prolonged hospitalization, invasive medical procedures, and exposure to antibiotics. In addition, MRSA can spread rapidly within healthcare settings, posing a significant threat to vulnerable patient populations. To address this issue, healthcare facilities must implement effective infection control measures, such as hand hygiene, environmental cleaning, and surveillance of MRSA [11].

Worryingly, many of these strains have developed multidrug resistance and are associated with high morbidity and mortality rates [12,13,14,15], which points to the urgent need to discover new antibacterial drugs capable of overcoming the antibiotic-resistance mechanism developed by these strains.

In this context, consistent evidence presents the bioprospecting of natural products as a promising alternative in the identification of bioactive compounds [16,17], extracts, and essential oils with the potential to be used in the development of new drugs for treating various diseases, including those caused by bacterial infections [18,19].

The utilization of plants for medicinal purposes is a longstanding tradition that has been transmitted through generations based on empirical knowledge. The medicinal properties of plants are attributed to the secondary metabolites, or phytochemicals, that they contain. These compounds not only provide therapeutic benefits but also protect the plants from pests and microbial infections [20].

The combination of antibiotics with nonantibiotic compounds is found to be a promising strategy in the combat of bacterial infections. Several studies have demonstrated the possibility of combining antibiotics with adjuvants or antimicrobial compounds, such as bioactive secondary metabolites of plants, also known as phytochemicals. Accordingly, evidence indicates that the essential oil substances are a group of phytochemicals with promising antibiotic-potentiating activity, as demonstrated by several in vitro studies [21].

Essential oils (EOs) are a blend of volatile compounds synthesized by aromatic plants that contain secondary metabolites. Therefore, essential oils are composed of secondary metabolites from plants. They serve a crucial function in safeguarding the plants from predators, microorganisms, and unfavorable weather conditions. The chemical structures of EOs are diverse and consist of various groups with distinct biosynthetic origins, including: 1-terpenes (monoterpenes and sesquiterpenes); 2-terpenoids (isoprenoids); 3-group with chemical characteristics of aliphatic; and 4-group with chemical characteristics of aromatic compounds (e.g., aldehydes, phenols). All of these groups are characterized by their low molecular weight. Monoterpenes are the primary components of EOs, and they have been found to exhibit potent antibacterial activity against microorganisms related to dental caries [22]. Essential oils have constituents, primarily terpenes, that can interact synergistically with antibiotics, enhancing their antibacterial effects. The phytochemical components of essential oils appear to facilitate the transport various compounds in the medium into the interior of the bacteria, thereby improving the antibacterial activity of drugs which acquire greater penetration capacity in the bacterial cell [23].

When antibacterials are combined with essential oils, their interaction can produce three possible results: synergistic, additive, or antagonistic. Synergy occurs when two antibacterial substances are combined, producing antibacterial activity that is greater than the summation of the antibacterial activity of each substance alone. An additive effect is produced when antibacterials are combined, resulting in an antibacterial effect that is the same the sum of the individual substances. Conversely, an antagonistic effect decreases the antibacterial activity of the two substances in combination compared to their individual antibacterial activity [24]. Essential oils have already demonstrated promising synergistic activity [25]. It appears that phytochemical found in essential oils favors the transportation of compounds to the interior bacteria, thereby improving the antibacterial efficacy of many drugs [20].

The Anacardiaceae family stands out for the considerable number of botanical species with medicinal properties [26,27,28]. Among these species, the plant *Schinus terebinthifolius* Raddi, commonly known in Brazil as “aroeira vermelha”, “aroeira mansa”, and “pimenta rosa” stands out, and is perceived in the Cerrado, Caatinga, Atlantic Forest and Pampas biomes. This species has a variety of applications, being used in folk medicine, in the ornamentation of large urban centers, and even for food purposes. In traditional medicine, it is used to prepare teas, baths, soaps, balms, and ointments. Therefore, it can be used to treat respiratory infections, fever, rheumatism, muscle, and intestinal problems, among other purposes. In this regard, phytochemical studies with the species have revealed that its constituents may have a bioactive action since these studies indicate the presence of phenols, flavonoids, steroids, triterpenes, anthraquinones, and saponins. Previous studies have shown that this species has anti-inflammatory, antimicrobial, anti-rheumatic, and healing activities [29,30]. Therefore, to expand scientific knowledge of the biological activities of *Schinus terebinthifolius* and to increase its possibility of becoming a potential herbal medicine, new studies are needed on its chemical characteristics, combined with detailed studies of its potential biological activities, with emphasis on antimicrobial activities.

Therefore, the present study aimed to evaluate the chemical composition and antibacterial activity of *Schinus terebinthifolius* Raddi essential oil (STEO) against standard and multidrug resistant (MDR) bacterial strains.

## 2. Results

The STEO extraction was achieved with a yield of 0.44%. The GC/MS analysis allowed the identification of 28 constituents, including alpha-pinene (24.3%), gamma-muurolene (16.6%), and myrcene (13.7%) as major compounds. Concerning the terpene classes identified in the STEO, it is noteworthy that sesquiterpenes (47.8%), monoterpenes (34.4%), oxygenated sesquiterpenes (11.8%), and oxygenated monoterpenes (1.1%) are predominant in the essential oil constitution, as shown in Table 1.

**Figure 1:**
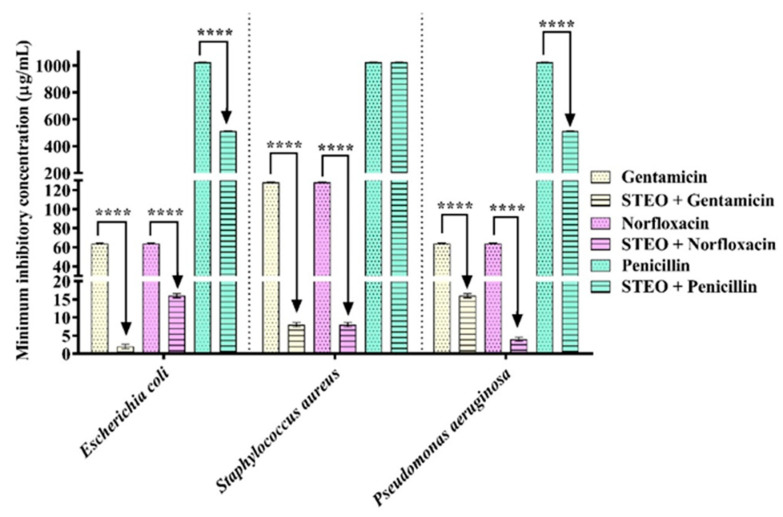
Antibiotic-enhancing effect of Schinus terebinthifolius essential oil (STEO) against MDR strains. **** (*p* < 0.0001).

Despite not having demonstrated intrinsic antibacterial activity (all with MIC of ≥1024 µg/mL), the STEO reduced the MIC of antibiotics such as gentamicin and norfloxacin, indicating the ability to potentiate the action of antibiotics against Gram-positive and Gram-negative strains (Figure 1). From a more careful analysis of this effect, it is possible to observe that the association with STEO caused a reduction of 50 to 96.87% in the MIC of antibiotics. STEO potentiated the action of gentamicin against all multidrug-resistant bacteria tested, being more effective against *E. coli* 06, where the MIC was reduced from 64 µg/mL to 2 µg/mL. In the case of norfloxacin, the MIC was decreased by 93.75% against *P. aeruginosa* 24 (from 64 to 4 µg/mL) and *S. aureus* 10 (128 to 8 µg/mL). The oil also potentiated the action of penicillin against *E. coli* 06, reducing, in both cases, the MIC of the antibiotic from 1024 to 512 µg/mL (50% reduction) (Figure 1).

## 3. Discussion

The biological activity of essential oils is determined by a variety of factors, such as the composition, types of functional groups of the active components, and their synergistic interactions [31]. The antibacterial activity of any essential oil depends on the relative abundance of biologically active constituents in the entire oil. However, the abundance of the main active constituents in essential oils is one of many factors responsible for their inherent activity since the interactions between these and minor constituents are also critical. In this context, previous research has demonstrated that combining eugenol with linalool or menthol exhibited significant synergism, suggesting that monoterpenoid alcohol is effective in potentiating their biological effects [32]. Testing of compounds or fractions of essential oils in combinations of two or four components has resulted in the identification of several synergistic antimicrobial activities [31].

The mechanism of action for antimicrobials varies depending on the type of essential oil (EO) and microbial strain. It is well known that Gram-positive bacteria are more susceptible to EOs than Gram-negative bacteria [25]. This can be attributed to the fact that Gram-negative bacteria have a rigid outer membrane which has a large amount of lipopolysaccharides (LPS). This membrane is more complex decreases diffusion of hydrophobic compounds through the plasma membrane. Conversely, Gram-positive bacteria are surrounded by a thick peptidoglycan wall with insufficient density to prevent the passage of small molecules, allowing for easier access to the cell interior [33,34].

Furthermore, the hydrophobic portions of lipoteichoic acid located within the cell membrane of Gram-positive bacteria could potentially aid in the penetration of hydrophobic constituents found in essential oils [35]. Therefore, essential oils can break the plasma membrane and the cell wall, increasing the penetrability in bacterial cell, causing the leakage of cytosol components, especially losses of electrolytes such as K+, Ca2+, Na+, and also losses of sugars and other molecules such as proteins and nucleic acids [36]. However, the present study failed to show direct antibacterial activity against Gram-positive and Gram-negative bacteria. When antibiotics were associated with essential oil, the effects were more significant against Gram-positive bacteria using antibiotics that act directly inside the cell. It is suggested that the membrane alteration allows the penetration of greater antibiotic doses inside the bacterial cell.

The results of the present study are corroborated by the findings of Uliana [37]. They identified 32 chemical constituents in the essential oil of *S. terebentifolius* leaves, including myrcene (6.78%) and alpha-pinene (4.05%) as significant constituents. Ennigrou et al. [38] studied the chemical composition of the essential oil of the leaves and branches of *S. terebinthifolius*, demonstrating the predominance of alpha-phellandrene (33.06–36.18%), alpha-pinene (14.85–15.18%), and limonene (6.62–8.79%) in leaves and branches, respectively.

The results obtained in the present study showed that STEO did not present significant antibacterial activity against the standard and multidrug-resistant strains of *E. coli*, *P. aeruginosa*, and *S. aureus* since this essential oil presented Minimum Inhibitory Concentration (MIC) values ≥1024 µg/mL. A MIC of this magnitude is considered irrelevant for clinical application since a high product concentration is required to perform direct antibacterial activity, implying a higher toxicity index [39].

On the other hand, a study by Cole et al. [40] evaluated the antibacterial activity of the essential oil of the ripe fruit of *S. terebinthifolius* against wild strains of hospital origin, including *E. coli*, *P. aeruginosa*, and *S. aureus*, demonstrating significant activity against all the strains, especially against the Gram-positives. Similarly, Salem et al. [41] explained that essential oil from ripe fruits of *S. terebinthifolius* has antibacterial activity against *S. aureus* and *P. aeruginosa*. Furthermore, Gundidza and collaborators [42] showed that essential oil obtained from fresh leaves of the same species showed activity against Gram-negative bacteria *E. coli* and *P. aeruginosa*.

Other species of the genus *Schinus* have also had their antibacterial properties previously demonstrated. In this perspective, El-Nashar et al. [43] verified the antibacterial activity of oil from leaves and bark of *Schinus polygamus* (Anacardiaceae) against *E. coli* ATCC 25922, *P. aeruginosa* ATCC 10145, *S. aureus* ATCC 33591, and two clinical isolates of this species. These authors demonstrated that while the leaf oil showed inhibitory action against all bacterial strains, the bark oil was effective only against *E. coli* ATCC 25922 and *P. aeruginosa* ATCC 10145.

On the other hand, the essential oil did not affect the resistance profile to the penicillin action observed in the tests with *S. aureus*. In this case, the resistance mechanism involves the production of penicillinases, which promotes the hydrolysis of the beta-lactam ring of penicillin, which causes its inactivation [44].

The pharmacological action demonstrated by STEO in the present study may be mediated by constituents such as alpha-pinene since previous studies have shown that this compound acts as a potentiator of the action of antibiotics. In addition, another significant component of the essential oil, alpha-pinene, potentiated the activity of norfloxacin against strains of *S. aureus* [24,45], *E. coli*, and *P. aeruginosa* [45], corroborating the findings of this study.

## 4. Materials and Methods

### 4.1. Botanical Material

Terminal branches and inflorescences of 10 specimens (approximately 1 kg) of *S. terebinthifolius* were collected in the municipality of Curitiba at the following coordinates: 25°19.982′ S, 49°48.351′ W, 1018 m altitude. The collection and transport of plant material were authorized by the Environment Institute of the State of Paraná (protocol number 284/11). After collection, voucher specimens were prepared and registered in the Herbarium of Faculdades Integradas Espírita- HFIE (registry number HFIE 8.814).

### 4.2. Essential Oil Extraction and Characterization

The essential oil was obtained by means of hydrodistillation using a Clevenger-type apparatus, referred to as STEO. In total, three extraction cycles were performed, using 1 L of water for every 50 g of dry leaves, with an overall extraction time of 150 min. To determine the yield of the essential oil, the dry weight of the botanical material used in the extraction was compared to the total mass of essential oil obtained on a dry basis. The chemical composition of the oil was analyzed using Gas Chromatography coupled with Mass Spectroscopy. Identification of chemical constituents was based on the comparison of their mass spectra with the Wiley and NIST library, as well as the analysis of linear retention indices. These indices were calculated by injecting a homologous series of hydrocarbons (C7–C26) and compared to previously published data. Only peaks with a concentration greater than 1% were considered for the identification and quantification of chemical components. The mass detector was operated in electron ionization mode (70 eV) at a scan rate of 3.15 min^−1^ and a mass range of 40–450 u, while the transfer line, ion source, and quadrupole analyzer were maintained at 260 °C, 230 °C, and 150 °C, respectively. For quantification, diluted samples were injected into an Agilent 7890A chromatograph equipped with a flame ionization detector (FID) operated at 280 °C, using the same column and analytical conditions as described above, with hydrogen as the carrier gas flowing at a rate of 1.5 mL min^−1^. The percentage of each component was determined through electronic integration of the FID signal by dividing the area of each component by the total area (area%) as per the methodology outlined by Adams [46].

### 4.3. Bacterial Strains

The standard bacterial strains used in this study were *E. coli* ATCC 25922, *P. aeruginosa* ATCC 9027, *S. aureus* ATCC 25923, while the MDR strains were *E. coli* 06, *P. aeruginosa* 24, and *S. aureus* 10. The origin and profile of resistance of these strains were described by Bezerra et al. [47], as shown in Table 2. These strains were stored at −20 °C.

### 4.4. Bacterial Inocula

Bacterial inocula were prepared in BHI (brain–heart infusion) broth from the cultured and grown bacterial colonies. Following the incubation period, cultures of the cultured bacteria were transferred to sterile test tubes containing 5 mL of isotonic saline (0.9% NaCl). The tubes holding the suspensions were agitated using a vortex mixer, and the cloudiness was standardized to 0.5 units on the McFarland scale.

### 4.5. Drugs and Reagents

Aliquots of 10 mg of STEO and antibiotics (gentamicin, norfloxacin, and penicillin) were weighed, dissolved in 1 mL of dimethylsulfoxide (DMSO), and diluted in 8765 μL of sterile distilled water to achieve the concentration of 1024 μg/mL. These drugs were obtained from SIGMA Chemical Co. (St. Louis, MO, USA).

### 4.6. Minimum Inhibitory Concentration (MIC) Determination

The STEO MIC was determined using the methodology described by Javadpour et al. [48]. Briefly, microtubes were spiked with 900 µL of 10% BHI medium and 100 µL of inoculum. Then, 100 µL of this solution was transferred to wells in a 96-well plate. After filling the plates, 100 µL of STEO was added to the first well of the plate, followed by serial dilution, reaching concentrations between 1024 µg/mL and 0.5 µg/mL. Wells in which the essential oil was not added were used as bacterial growth control. The plates were incubated in a bacteriological oven at a temperature of 37 °C, and after 24 h, the wells were added with 20 μL of sodium resazurin. The results were read 1 h later by observing the color change in the wells, where the pink color indicates bacterial growth. In contrast, the permanence of the blue color represents growth inhibition. All tests were performed in triplicate.

### 4.7. Analysis of Antibiotic Activity Modulation

Following the methodology proposed by Coutinho et al. [49], Eppendorf-type microtubes were filled with 150 µL of bacterial inoculum, 10% BHI medium, and STEO in a volume sufficient to reach a subinhibitory concentration of the essential oil (equivalent to MIC/8), reaching a final volume of 1350 µL. Control tubes were filled with 1350 µL of 10% BHI medium and 150 µL of bacterial inoculum. The contents of the tubes were distributed into the wells of a 96-well plate in a volume of 100 µL per well. Then, a serial dilution was performed with 100 µL of each antibiotic, reaching concentrations between 1024 µg/mL and 0.5 µg/mL. Bacterial growth analysis was performed as described previously. All tests were performed in triplicate.

### 4.8. Statistical Analysis

Statistical analysis was performed using the GraphPad Prism statistical program version 9.0. The means were analyzed by two-way analysis of variance (ANOVA) followed by Bonferroni’s post hoc test, where a *p* < 0.05 was considered significant.

## 5. Conclusions

The present research revealed that STEO is chemically characterized by bioactive compounds such as alpha-pinene, gamma-muurolene, and myrcene. However, this essential oil showed no significant antibacterial activity since it presented clinically irrelevant MIC values against standard *E. coli*, *P. aeruginosa*, and *S. aureus* ATCC 25923.

Notably, the association of essential oil with gentamicin and norfloxacin potentiated antibiotic activity against Gram-positive and Gram-negative strains. In addition, the action of penicillin against Gram-negative bacteria was also enhanced by STEO.

From this research, it can be inferred that STEO has significant antibiotic-potentiating activity and can be a promising source of new molecules to combat antibiotic resistance.

## Figures and Tables

**Table 1:** The chemical composition of *Schinus terebinthifolius* Raddi essential oil.

Constituent	%	RI *	Constituent	%	RI *
alpha-pinene	24.3	937	(E)-beta-farnesene + allo-aromadendrene	0.9	1457
alpha-fenchene	0.3	951	gama-muurolene	16.6	1478
sabinene	1.0	975	Bicyclogermacrene	3.8	1492
beta-pinene	4.1	979	alpha-muurolene	0.5	1495
myrcene	13.7	991	germacrene A	1.1	1499
para-cymene	0.4	1025	delta-cadinene	1.8	1519
limonene	1.5	1030	germacrene B	0.6	1551
(*Z*)-beta ocimene	0.3	1039	Spathulenol	1.7	1573
terpinen-4-ol	0.9	1176	caryophyllene oxide	2.1	1577
alpha-terpineol	0.9	1189	Viridiflorol	0.6	1585
alpha-copaene	3.8	1373	cubeban-11-ol	0.4	1587
beta-elemene	1.6	1389	*epi*-alpha-cadinol	1.7	1637
(*E*)-caryophyllene	8.9	1416	alpha-muurolol	0.6	1641
alpha-humulene	1.1	1449	alpha-cadinol	2.3	1649

* Retention index = Retention time compared with authentic compounds.

**Table 2:** Origin and resistance profile of the MDR strains selected for the study.

Strain	Origin	Resistance Profile
*E. coli* 06	Urine	Asb, Ca, Cef, Cfo, Cpm, Cro
*P. aeruginosa* 24	Nasal Discharge	Ami, Cip, Cpm, Ctz, Imi, Lev, Mer, Ptz
*S. aureus* 10	Rectal Swab	Oxa, Pen

Legend: Ami—Amikacin, Asb—Ampicillin + Sulbactam, Ca—Cefadroxil, Cef—Cephalexin, Cfo—Cefoxitin, Cip—Ciprofloxacin, Cpm—Cefepime, Cro—Ceftriaxone, Ctz—Ceftazidime, Imi—Imipenem, Lev—Levofloxacin, Mer—Meropenem, Oxa—Oxacillin, Pen—Penicillin, Ptz—Piperacillin + Tazobactam.

## Data Availability

Not applicable.
